# Complete genome sequence of *Streptococcus agalactiae* strain SA20-06, a fish pathogen associated to meningoencephalitis outbreaks

**DOI:** 10.4056/sigs.3687314

**Published:** 2013-05-25

**Authors:** Ulisses de Pádua Pereira, Anderson Rodrigues dos Santos, Syed Shah Hassan, Flávia Figueira Aburjaile, Siomar de Castro Soares, Rommel Thiago Jucá Ramos, Adriana Ribeiro Carneiro, Luís Carlos Guimarães, Sintia Silva de Almeida, Carlos Augusto Almeida Diniz, Maria Silvanira Barbosa, Pablo Gomes de Sá, Amjad Ali, Syeda Marriam Bakhtiar, Fernanda Alves Dorella, Adhemar Zerlotini, Flávio Marcos Gomes Araújo, Laura Rabelo Leite, Guilherme Oliveira, Anderson Miyoshi, Artur Silva, Vasco Azevedo, Henrique César Pereira Figueiredo

**Affiliations:** 1 AQUAVET- Laboratory of Aquatic Animal Diseases, Department of Preventive Veterinary Medicine, Federal University of Minas Gerais, Belo Horizonte, MG, Brazil; 2 Institute of Biologic Sciences, Federal University of Minas Gerais, Belo Horizonte, MG, Brazil; 3 Institute of Biologic Sciences, Federal University of Pará, Belém, PA, Brazil; 4 Department of Veterinary Medicine, Federal University of Lavras, Lavras, MG, Brazil; 5 Center for Excellence in Bioinformatics - FIOCRUZ-MG, Belo Horizonte, MG, Brazil; 6 Bioinformatics Multiuser Laboratory - Embrapa, Campinas, SP, Brazil

**Keywords:** *Streptococcus agalactiae*, fish pathogen, genome sequencing

## Abstract

*Streptococcus agalactiae* (Lancefield group B; GBS) is the causative agent of meningoencephalitis in fish, mastitis in cows, and neonatal sepsis in humans. Meningoencephalitis is a major health problem for tilapia farming and is responsible for high economic losses worldwide. Despite its importance, the genomic characteristics and the main molecular mechanisms involved in virulence of *S. agalactiae* isolated from fish are still poorly understood. Here, we present the genomic features of the 1,820,886 bp long complete genome sequence of *S. agalactiae* SA20-06 isolated from a meningoencephalitis outbreak in Nile tilapia (*Oreochromis niloticus*) from Brazil, and its annotation, consisting of 1,710 protein-coding genes (excluding pseudogenes), 7 rRNA operons, 79 tRNA genes and 62 pseudogenes.

## Introduction

*Streptococcus agalactiae,* also referred as Group B *Streptococcus* (GBS), is a Gram-positive pathogen with a broad host range. GBS is the most common cause of life-threatening bacterial infections in human newborns [1] and is an important etiological agent of clinical and sub-clinical bovine mastitis [2]. In fish, *S. agalactiae* infection causes septicemia and meningoencephalitis, mainly in warm water species from freshwater, marine, or estuarine environments [3]. Currently, *S. agalactiae* is an emerging pathogen associated with severe economic losses due to high mortality rates in fish farms worldwide [4,5].

The pangenome of the species (obtained from only eight human strain genomes) is considered open and it is expected that, for every new GBS genome sequenced, approximately 33 new strain-specific genes will be identified [6]. Since, the first genome of *S. agalactiae* strain isolated from bovine mastitis was published and 183 strain-specific genes were described, and about 85% of these genes have been clustered into eight genome islands, strongly suggesting that these genes were acquired through lateral gene transfer from other bacteria of genus *Streptococcus*, which are also etiologic agents of bovine mastitis [2]. However, the molecular mechanisms of virulence and other genomic features of strains isolated from fish isolates remain unclear, and thus, the genome sequencing of different strains isolated from other hosts are still required to better understand the global complexity of this bacterial species.

## Classification and Features

The genus *Streptococcus* comprises a heterogeneous group of bacteria that have an important role in medicine and industry. These microorganisms are Gram-positive, cocci, 0.6-1.2 µm diameter, not motile, do not form spores, are catalase-negative and grow in pairs or chains [7]. Rebecca C. Lancefield, in her work in the early 1930s, systematized the classification of streptococci based on the presence and type of surface antigen: cell wall polysaccharide or lipoteichoic acid [8]. *S. agalactiae* is classified as Lancefield group B (GBS) based on the presence of a polysaccharide in the cell wall. This polysaccharide is composed of galactose, N-acetylglucosamine, rhamnose and glucitol phosphate [7].Currently, ten serotypes are described for this species (Ia, Ib, II-IX) and occasionally some strains can be non-serotypeable [9].

Major human and animal streptococcal pathogens belong to the pyogenic group of β-hemolytic streptococci [10]. In this context, the β- hemolytic bacteria *S. agalactiae*, deserves attention for causing diseases in a broad range of homeothermic and heterothermic hosts [4], although this bacteria is also a common member of the gastrointestinal tract microbiota [11].

At the end of the 19th century, GBS was initially described as an etiological agent of mastitis in cows, being reported as causing disease in humans only 50 years later [12]. In fish, *S. agalactiae* was recognized as a pathogen in 1966 [13]. Sporadically, this pathogen has also been associated with illness in many others hosts, such as chickens, camels, dogs, horses, cats, frogs, hamsters, mice, monkeys, and nutria [14].

*S. agalactiae* is a facultatively anaerobic bacterium that uses glucose as an energy source, and is also able to use different carbon sources such as cellobioise, beta-glucoside, trehalose, mannose, lactose, fructose, mannitol, N-acetylgalactosamine, and glucose (Table 1). This pathogen is limited in the synthesis of most amino acids precursors. Only the biosynthetic pathways for alanine, serine, glycine, glutamine, aspartate, asparagine and threonine are present [31]. The adaptation to oxygen radical stress of this pathogen is related to superoxide dismutase (*sodA* gene) which converts superoxide anions to molecular oxygen and hydrogen peroxide, which, in turn, is metabolized by catalases and/or peroxidases [34]. Although GBS does not synthetize catalase to remove toxic H_2_O_2_, it is 10-fold more resistant to oxygen metabolites than the catalase-producing *S. aureus*. This is due to the presence of several enzymes that might detoxify H_2_O_2_ that have been identified in the genome of *S. agalactiae* such as NADH peroxidase, NADH oxidase and thiol peroxidase [31]. This diversity of metabolic and adaptative mechanisms reflects the ability of GSB to survive in various environments and hosts.

**Table 1:** Classification and general features of *S. agalactiae* SA20-06 according to the MIGS recommendations [15].

**MIGS ID**	**Property**	**Term**	**Evidence code**
	Classification	Domain *Bacteria*	TAS [16]
		Phylum *Firmicutes*	TAS [17-19]
		Class *Bacilli*	TAS [20,21]
		Order *Lactobacillales*	TAS [20,22]
		Family *Streptococcaceae*	TAS [23,24]
		Genus *Streptococcus*	TAS [23,25,26]
		Species *Streptococcus agalactiae*	TAS [23,27-29]
		Strain SA20-06	TAS [4]
	Gram stain	Positive	TAS [30]
	Cell shape	Spherical or ovoid	TAS [30]
	Motility	non-motile	TAS [30]
	Sporulation	non-sporulating	TAS [30]
	Temperature range	mesophile	TAS [30]
	Optimum temperature	28°C (fish isolates)	IDA
	Salinity	usually grows in 4% of NaCl, but not in 6.5%	TAS [30]
MIGS-22	Oxygen	Facultative anaerobe	TAS [30]
	Carbon source	cellobioise, beta-glucoside, trehalose, mannose, lactose, fructose, mannitol, N-acetylgalactosamine, and glucose	TAS [31]
	Energy source	Chemoorganotroph with fermentative metabolism	TAS [30]
MIGS-6	Habitat	Host	TAS [4]
MIGS-15	Biotic relationship	Symbiotic (pathogen)	TAS [4]
MIGS-14	Pathogenicity	Cows, human, fishes and other animals	TAS [12,14]
	Biosafety level	2	TAS [32]
	Isolation	Kidney of Nile tilapia	TAS [4]
MIGS-4	Geographic location	Parana state, Brazil	TAS [4]
MIGS-5	Sample collection time	2006	TAS [4]
MIGS-4.1 MIGS-4.2	Latitude Longitude	not reported not reported	
MIGS-4.3	Depth	not reported	
MIGS-4.4	Altitude	not reported	

Evidence codes - IDA: Inferred from Direct Assay; TAS: Traceable Author Statement (i.e., a direct report exists in the literature); NAS: Non-traceable Author Statement (i.e., not directly observed for the living, isolated sample, but based on a generally accepted property for the species, or anecdotal evidence). These evidence codes are from the Gene Ontology project [33]. If the evidence is IDA, then the property was directly observed for a live isolate by one of the authors or an expert mentioned in the acknowledgements.

**Figure 1:**
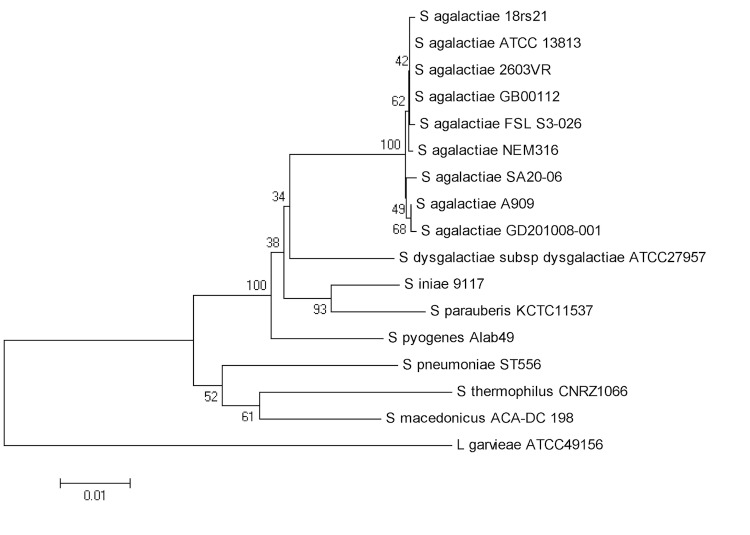
Phylogenetic tree highlighting the position of *S. agalactiae* strain SA20-06 in relation to other selected strains of the species and others from the genus *Streptococcus*. The tree was based on 1,410 characters of the 16S rRNA gene sequence aligned using ClustalW2 [35]. The tree was inferred under the maximum likelihood criterion using MEGA5 software [36] and rooted with 16S rRNA sequence of fish pathogen *Lactococcus garvieae* (a member of the *Streptococcaceae*). The branches were mapped by the expected number of substitutions per site. The numbers above the branches are support values from 1,000 bootstrap replicates. The strains and their corresponding GenBank accession numbers (and, when applicable, draft sequence coordinates) for 16S rRNA genes are: *S. agalactiae* 18rs21, NZ_AAJO01000124; *S. agalactiae* ATCC13813, NR_040821; *S. agalactiae* 2603VR, NC_004116; *S. agalactiae* GB00112, AKXO01000029; *S. agalactiae* FSL_S3-026, AEXT01000002; *S. agalactiae* NEM316, AL766845; *S. agalactiae* SA20-06, NC_019048; *S. agalactiae* A909, NC_007432; *S. agalactiae* GD201008-001, CP003810; *S. dysgalactiae subsp dysgalactiae* ATCC 27957, CM001076; *S. iniae* 9117, NZ_AMOO01000003; *S. parauberis* KCT 11537, NC_015558; *S. pyogenes* alab49, NC_017596; *S. pneumoniae* ST556, NC_017769; *S. thermophilus* CNRZ1066, NC_006449; *S. macedonicus* ACA-DC 198, NC_016749; *L. garvieae*, AP009332.

The phylogenetic tree was constructed using 16S rRNA sequences of available *S. agalactiae* genomes and other species from the same genus (Figure1). The tree shows that all *S. agalactiae* strains are grouped together, and the SA20-06 strain is more similar to the A909 human isolate and to the GD201008-001 fish isolate from China.

## Genome sequencing and annotation

### Genome project history

This strain was selected for sequencing based on the high mortality rates shown for this pathogen in fish farms worldwide and on the lack of information for the genomic characteristics of *S. agalactiae* isolated from fish and the molecular mechanisms involved in virulence in this host. The genome project is deposited in the Genomes On Line Database [37] and the *Streptococcus agalactiae* SA20-06 complete genome sequence and annotation data were deposited in the DDBJ/EMBL/GenBank under the accession number CP003919 (RefSeq NC_019048). Sequencing, assembly steps, finishing and annotation were performed by the teams from the Laboratory of Cellular and Molecular Genetics (LGCM), Minas Gerais, Brazil; Genomics and Proteomics Network of the State of Pará (RPGP), Pará, Brazil and Center for Excellence in Bioinformatics (CEBio-FIOCRUZ-MG), Minas Gerais, Brazil. A summary of the project information is shown in Table 2.

**Table 2:** Genome sequencing project information.

**MIGS ID**	**Property**	**Term**
MIGS-31	Finishing quality	Finished
MIGS-28	Libraries used	Two mate-paired libraries (mean size 50 or 60 bp, DNA insert size of 1-2Kb)
MIGS-29	Sequencing platforms	SOLiD v3 plus and SOLiD 5500
MIGS-31.2	Sequencing coverage	5700×
MIGS-30	Assemblers	CLC Genome Workbench, Velvet, Edena
MIGS-32	Gene calling method	Glimmer
	Genbank ID	CP003919 (chromosome)
	Genbank Date of Release	November 02, 2012
	GOLD ID	Gc02347
	Project relevance	Animal and human pathogen

### Growth conditions and DNA isolation

*Streptococcus agalactiae* SA20-06 was obtained from the AQUAVET (Laboratory of Aquatic Animal Diseases) bacterial collection, streaked onto 5% sheep blood agar and incubated at 28^o^C for 48 h. After that, cells were grown in 150mL brain-heart-infusion broth (BHI-HiMedia Laboratories Pvt. Ltda, India) under agitation (150 rpm), at 28^o^C. Genomic DNA was obtained by using phenol-chloroform-isoamylic alcohol extraction protocol using micro-wave oven [38].

### Genome sequencing and assembly

The genome sequencing of *S. agalactiae* SA20-06 was performed using the SOLiD v3 Plus and SOLiD 5500 platforms (Applied Biosystems) with two mate-paired libraries (both with 1-2 kb insert size), which generated 50,223,637 and 283,953,694 reads of 50 bp and 60 bp in size, respectively. After sequencing, the reads were subjected to quality filtering using the qualityFilter.pl script (a homemade script), in which reads with an average Phred quality of less than 20 were removed, and error sequence correction was performed with SAET software (Life Technologies).

After quality analysis, 210,004,694 reads were used in the assembly, which generated a genome coverage corresponding to ~5,700× genome coverage based on the reference genome of 2,127,839 bp size of *S. agalactiae* strain A909 (NC_007432). The genome sequence of SA20-06 was assembled based on the hybrid strategy using CLC Genome Workbench 4.9, Velvet [39] and Edena [40] software. A total of 872 contigs were generated, with *N*_50_ of 5,221 bp and the smallest contig having 201 bp. Due to the hybrid assembly methodology, the redundant contigs were removed using the Simplifier software [41]. The contigs were mapped against the reference genome (strain A909) using BLASTn, and the results were analyzed using G4ALL software [42], to extend the contigs and identify overlaps of a minimum of 30 bp between the ends of the contigs, thus yielding larger contigs.

These contigs were later subjected to a finishing process using CLC Genomics Workbench software. At this step, the contigs were ordered and oriented by mapping against the reference genome, yielding a preliminary scaffold with gaps that were removed with recursive rounds of short read mapping against the scaffold [43].

### Genome annotation

For structural annotation, the following software was employed: Glimmer 3, to predict genes [44]; RNAmmer, to predict rRNAs [45]; and tRNAscan-SE, to predict tRNAs [46]. Functional annotation was performed by similarity analyses using public databases of National Center for Biotechnology Information (NCBI) non-redundant database, Swiss-Prot and InterProScan analysis [47]. Genome visualization and manual annotation were carried out using Artemis [48].

## Genome properties

**Figure 2:**
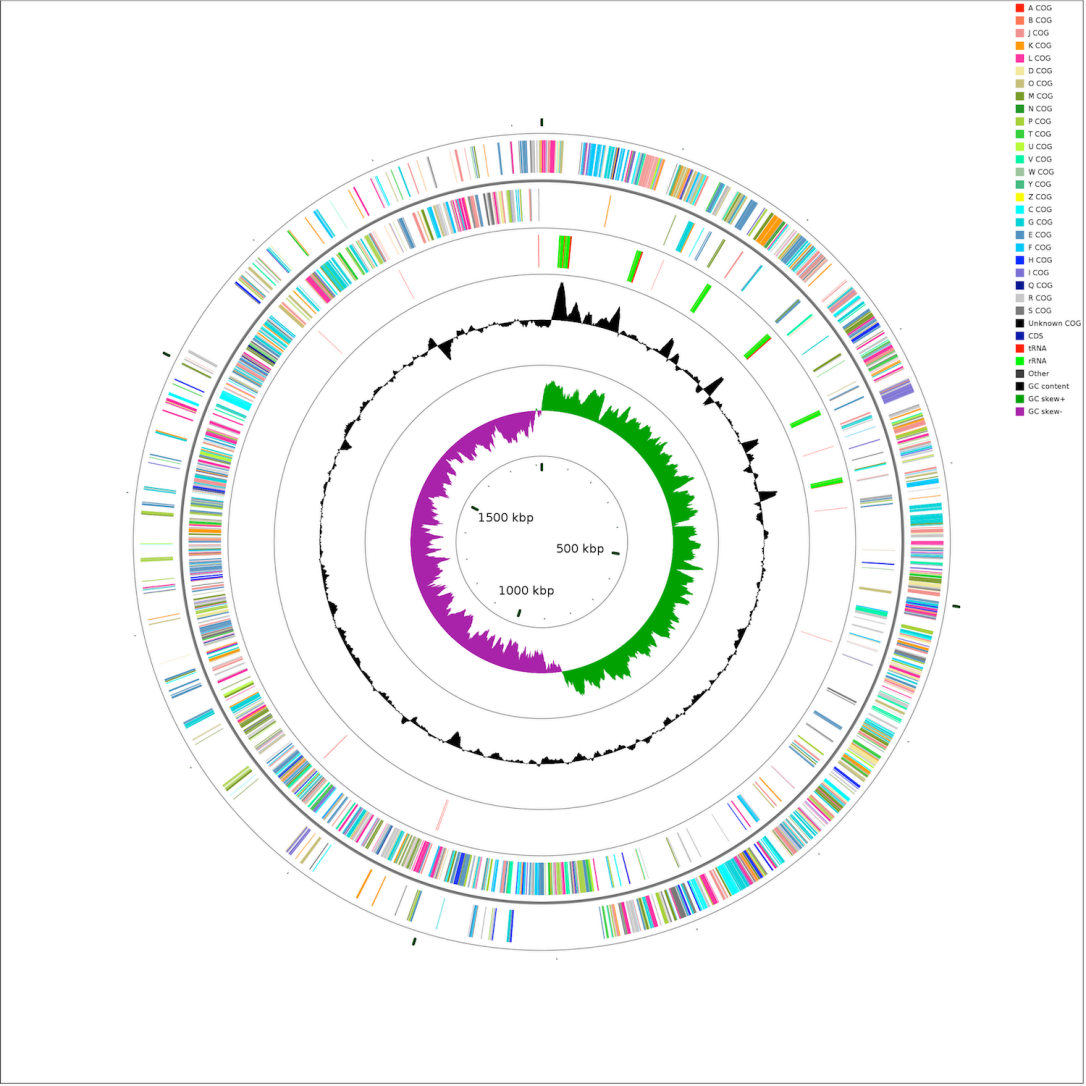
Graphical circular map of the genome performed with CGview comparison tool [49]. From outer to inner circle: Genes on forward strand (color by COG categories), Genes on reverse strand (color by COG categories), RNA genes (tRNAs red, rRNAs green, other RNAs black), GC content, GC skew.

The complete genome of *S. agalactiae* strain SA20-06 comprises a single circular chromosome of 1,820,886 bp in length with 1,710 putative predicted genes (excluding pseudogenes), 35.56% G+C content, 7 rRNA operons, 79 tRNA genes and 62 pseudogenes (Figure 2 and Table 3). The distribution of genes into the COG functional categories is presented in Table 4.

**Table 3:** Genome Statistics.

**Attribute**	**Value**	**% of Total^a^**
Genome size (bp)	1,820,886	100.00%
DNA coding region (bp)	1,547,993	85.01%
DNA G+C content (bp)	647,477	35.56%
Number of replicons	1	
Extrachromosomal elements	0	
Total genes^b^	1,872	100.00%
RNA genes	100	5.34%
rRNA operons	7	
Protein-coding genes	1,772	94.66%
Pseudo genes	62	3.31%
Genes with function prediction	1,515	80.93%
Genes in paralog clusters	430	22.97%
Genes assigned to COGs	1,469	78.47%
Genes assigned Pfam domains	1,547	82.64%
Genes with signal peptides	302	16.13%
Genes with transmembrane helices	447	23.88%

a) The total is based on either the size of the genome in base pairs or the total number of protein coding genes in the annotated genome.

b) Also includes 62 pseudogenes.

**Table 4:** Number of genes associated with the general COG functional categories.

**Code**	**Value**	**%age**	**Description**
J	146	9.2	Translation, ribosomal structure and biogenesis
A	0	0.0	RNA processing and modification
K	118	7.44	Transcription
L	86	5.42	Replication, recombination and repair
B	0	0.0	Chromatin structure and dynamics
D	17	1.07	Cell cycle control, cell division, chromosome partitioning
Y	0	0.0	Nuclear structure
V	36	2.27	Defense mechanisms
T	66	4.16	Signal transduction mechanisms
M	92	5.8	Cell wall/membrane biogenesis
N	6	0.38	Cell motility
Z	0	0.0	Cytoskeleton
W	0	0.0	Extracellular structures
U	21	1.32	Intracellular trafficking and secretion
O	53	3.34	Posttranslational modification, protein turnover, chaperones
C	46	2.9	Energy production and conversion
G	150	9.45	Carbohydrate transport and metabolism
E	134	8.44	Amino acid transport and metabolism
F	75	4.73	Nucleotide transport and metabolism
H	52	3.28	Coenzyme transport and metabolism
I	43	2.71	Lipid transport and metabolism
P	86	5.42	Inorganic ion transport and metabolism
Q	19	1.2	Secondary metabolites biosynthesis, transport and catabolism
R	192	12.10	General function prediction only
S	149	9.39	Function unknown
-	403	21.53	Not in COGs

## Conclusions

Further analysis of the SA20-06 genome is now under way, with the objective of identifing specific factors that might explain the differences in pathogenesis of disease, mainly in heterothermic hosts.
